# The Discharge Communication Study: research protocol for a mixed methods study to investigate and triangulate discharge communication experiences of patients, GPs, and hospital professionals, alongside a corresponding discharge letter sample

**DOI:** 10.1186/s12913-019-4612-1

**Published:** 2019-11-11

**Authors:** Katharine Weetman, Jeremy Dale, Emma Scott, Stephanie Schnurr

**Affiliations:** 1 Unit of Academic Primary Care, Division of Health Sciences, Warwick Medical School, University of Warwick, Coventry, CV4 7AL UK; 2 Centre for Applied Linguistics, University of Warwick, Coventry, CV4 7AL UK

**Keywords:** Communication, Discharge letter, Continuity of care, Hospital discharge, Mixed methods

## Abstract

**Background:**

Discharge letters are crucial during care transitions from hospital to home. Research indicates a need for improvement to increase quality of care and decrease adverse outcomes. These letters are often sent from the hospital discharging physician to the referring clinician, typically the patient’s General Practitioner (GP) in the UK, and patients may or may not be copied into them. Relatively little is known about the barriers and enablers to sending patients discharge letters. Hence, the aim of this study was to investigate from GP, hospital professional (HP) and patient perspectives how to improve processes of patients receiving letters and increase quality of discharge letters. The study has a particular focus on the impacts of receiving or not receiving letters on patient experiences and quality of care.

**Methods:**

The setting was a region in the West Midlands of England, UK. The research aimed to recruit a minimum of 30 GPs, 30 patients and 30 HPs in order to capture 90 experiences of discharge communication. Participating GPs initially screened and selected a range of recent discharge letters which they assessed to be successful and unsuccessful exemplars. These letters identified potential participants who were invited to take part: the HP letter writer, GP recipient and patient. Participant viewpoints are collected through interviews, focus groups and surveys and will be “matched” to the discharge letter sample, so forming multiple-perspective “quartet” cases. These “quartets” allow direct comparisons between different discharge experiences within the same communicative event. The methods for analysis draw on techniques from the fields of Applied Linguistics and Health Sciences, including: corpus linguistics; inferential statistics; content analysis.

**Discussion:**

This mixed-methods study is novel in attempting to triangulate views of patients, GPs and HPs in relation to specific discharge letters. Patient and practitioner involvement will inform design decisions and interpretation of findings. Recommendations for improving discharge letters and the process of patients receiving letters will be made, with the intention of informing guidelines on discharge communication. Ethics approval was granted in July 2017 by the UK Health Research Authority. Findings will be disseminated in peer-reviewed journals, reports and newsletters, and presentations.

## Background

The pressure on health services is heightening, in large part due to the need to cope with an increasingly multi-morbid and ageing population [1]. This means use of resources must be maximised, inefficiencies that are due to fragmented care or duplication of care need to be avoided, and patients need to be supported to self-care and self-manage more effectively. Effective communication and co-ordination between healthcare professionals and patients to facilitate positive outcomes is essential [2]. This is particularly key following care transitions, such as when discharging patients from hospital. The communication that takes place in relation to hospital discharge may be termed “discharge communication”.

In the UK, “discharge communication” may follow inpatient or outpatient discharge and typically takes written form as a discharge letter or summary sent from the discharging clinical team to the clinician who is to continue patient care, usually the General Practitioner (GP). Written discharge communication may be sent electronically or in hard copy; they may contain information relating but not limited to a summary of the patient’s hospital visit, treatment and required followed up. Such communication is generally described as being a “discharge letter” in the UK, and this is the term that we have used throughout the current paper. The content and structure of such discharge letters vary depending on the speciality, type of hospital care (e.g. outpatient or inpatient), and the individual preferences and style of the physician who authored the content.

Whilst in the UK patients receiving letters is considered ‘good practice’ [3, 4] and encouraged through initiatives [5] and guidelines [3, 4, 6], it is not standardised. Hence, patients may or may not receive these discharge communication letters [7, 8] but the reasons for this and subsequent effects remain unclear.

Previous research [9–11] indicates the quality and content of discharge letters may vary and does not always satisfy the requirements of those receiving them (e.g. GPs and/or patients). The factors influencing this variation and the extent of the subsequent impacts and effects on patients remain equivocal. Nonetheless, it is known that lack of care continuity through sub-standard discharge communication can lead to adverse outcomes [12, 13] such as preventable readmissions [14, 15]. Thus, ensuring high standards of discharge communication and improving current processes is important.

### Existing studies and narrowing the research focus

#### Scoping review

A scoping review was conducted to identify “themes” around problematic areas of discharge communication and whether further research is needed. Sources were initially searched up until August 2016. Publications and evidence were monitored thereafter. Health and Social Science journals were searched in addition to bibliographic databases, healthcare websites, government archives and grey literature. Pearling, hand-searching and ‘cited by’ searching were also undertaken. Previous relevant reviews [16–29] were consulted to inform the search strategy and review content. Research was contextualised by healthcare setting with a focus on the NHS.

A list of the primary scoping search terms can be found in Table 1; terms used were iteratively adapted for sources as required. Any evidence relevant to improvement of discharge communication was selected and included in the review (see Additional file 1 for list of included documents).

**Table 1:** List of search terms

1. Discharge communication	
2. Discharge summary(ies)	
3. Discharge letter(s)	
4. Discharge planning	
5. Secondary to primary care communication	
6. Patient discharge	
7. Hospital GP communication	
8. Hospital specialist discharge communication	
9. Information sent to GPs following discharge	
10. Discharge documents for GPs	
11. Hospital discharge letters	
12. Discharge documents	
13. Discharge information	
14. Hospital to primary care/family physician communication	
15. Discharge information for GPs	
16. Discharge information for family/primary care physicians	
17. Communication following patient discharge	
18. Discharge process	
19. Communication AND discharge	
20. Electronic discharge medicine information	
21. Integrated care information communication to GPs	
22. Hospital discharge information communication	

The scoping review identified multiple areas for further improvement. These included but were not limited to the areas listed in Table 2 which summarises the main findings.

**Table 2:** Summary of scoping review findings

Discharge communication area	Summary of main findings from scoping review
1. Mode, timing and medium of letters	Discharge letters are not always received by physicians in an adequate timeframe. Quality impacts and ethical and legal implications of technological interventions and affordances of electronic communication need further research.
2. Letter content	Discharge letters do not always contain sufficient detail relative to content components considered important to recipients e.g. diagnosis. Reasons for content variation, despite availability of guidelines, needs further research as well as better understanding of content items and details important to those involved in discharge communication.
3. Patients receiving letters	Patients receiving discharge letters, where there is no identified risk of this being harmful, is currently considered to be good practice. However, patients do not always receive letters. Reasons for this inconsistency and variation was unclear and needs further research. The format of patient letters vary, and include patient personalised letters and receiving a copy of the letter sent to the GP. The implications of these differing letter forms in terms of cost-benefit analysis and patient outcomes are indeterminate and require further research.
4. Letter form	A variety of letter forms may be used for discharge communication, such as dictated letter forms and structured discharge summary templates. Future research should assess feasibility and implications of interventions for integrating more standardised systems.
5. Letter authorship	There are potential issues with junior doctors and inexperienced practitioners producing discharge letters without adequate support. Support interventions such as training may increase discharge quality. Further research is needed to design, implement and evaluate feasible and sustainable training and support interventions.
6. Letter quality related to safety implications	Poor quality of discharge communication can pose risks to patient safety. Vulnerable groups such as those with medically complex needs, the elderly, those with low health literacy, and those with a lack of social or family support may be particularly at risk. Further research is needed to understand the needs of these groups and how risks to patient safety can be reduced through improved communication quality.
7. Medication information	Adequate details regarding medication information, particularly changes to medication, are not always included in discharge letters or clear to recipients. Further research should look at feasible and sustainable interventions for improving communication of medication information.

#### Patient and public involvement

Following the scoping review, patient and public involvement [30] (PPI) work was conducted with groups of patients and clinicians to inform the research design. Patients and clinicians were recruited through a variety of routes using the existing networks of the local Clinical Research Network (CRN), Warwick Medical School and collaborating Clinical Commissioning Groups (CCGs), patient participation groups (PPGs), and opportunistic and “snowball” methods. Involvement methods included a hard-copy and electronic survey and discussions with KW, either in groups, individually, or over the telephone.

Approximately 30 patients and 60 clinicians were involved in helping to identify research priorities [31] through “ranking” and commenting on potential areas of research identified from the scoping review. The main purpose was to increase the relevance and importance of the research to the NHS and needs of patients and clinicians [32, 33]. Through this process, the selected primary focus area for research questions was ‘patients receiving letters’ with ‘letter content’ selected as the secondary focus.

#### Realist review

Thereafter, a realist review [34] was undertaken which synthesised evidence for the intervention of ‘patients receiving letters’ in greater detail than had been possible in the scoping review. The full protocol for this review has been registered with Prospero (CRD42017069863) and published by BMJ Open [35]. The protocol [35] argues a realist synthesis is apt and useful as this approach has the capacity to account for *complexity* and theorise how an intervention may “work” (or not) [36, 37]*.* The intervention is complex in that the *form* of discharge communication can vary and the *quality* of communication is highly context-dependent. The review produced a resultant *programme theory* [37, 38] for the intervention. As outlined in the work of Pawson [38–43], a “programme theory” comprises a series of “context, mechanism, outcome” configurations and explains how an intervention or programme may be theorised to “work”; this details within what contexts, for whom, why and to what extent [36, 38, 39, 41].

The review [34] revealed discrepancies relating to benefits and drawbacks of the intervention and concluded that further research to better understand and explain outcome discrepancies is required. It also identified a need for further research to explore identified barriers to patients receiving discharge letters, such as clinician views.

### Rationale for research

Research is needed to improve the quality of discharge letters and the processes surrounding these letters, as well as better understanding of the impacts of current policies and guidelines. In particular, research is needed which both explores the reasons behind variation of patients receiving letters and the effects that this variation has on the patient’s experience and care. Such research needs to consider the perspectives of the patient, the physician writing the letter, and the physician who receives it, as all three perspectives are likely to influence the effectiveness and uptake of policies and guidelines. This study aims to address these needs through an innovative exploratory design to address research questions that centre on processes of patients receiving letters and discharge letter content and quality. The study is anticipated to provide further insights into the discrepancies that follow from whether or not patients receive letters [8, 44] and hence make recommendations for improving discharge communication processes. Findings and recommendations will be of direct and immediate relevance to GPs, hospital professionals, commissioners and policy makers, as well as service users.

### Research questions

The overarching aim of the study is to identify ways of improving written discharge communication between hospital healthcare professionals, GPs and patients. Research questions are:

1. In what form do patients currently receive discharge letters and why?
2. What are the effects of patients receiving written discharge letters?
3. Should patients receive or not receive discharge letters, why and in what form?
4. What are the features and key content-items of ‘successful’ discharge letters?

## Methods/design

### Study design

We will conduct an exploratory mixed methods three phase study over a 4-year period. The study is being undertaken to fulfil the requirements of a PhD, and started in September 2015. It is anticipated to continue until November 2019.

Three phases of data collection are planned. Phase I involves discharge letter sampling and exploration of the quality of discharge letters from the perspective of GPs. Phase II explores patient perspectives through interviews. Phase III considers hospital professional (HP) viewpoints through a survey. The study design aims for the phases to be sequential but allows for them to overlap to fit around needs of participants, the available research resources, and time limitations.

**Fig. 1:**
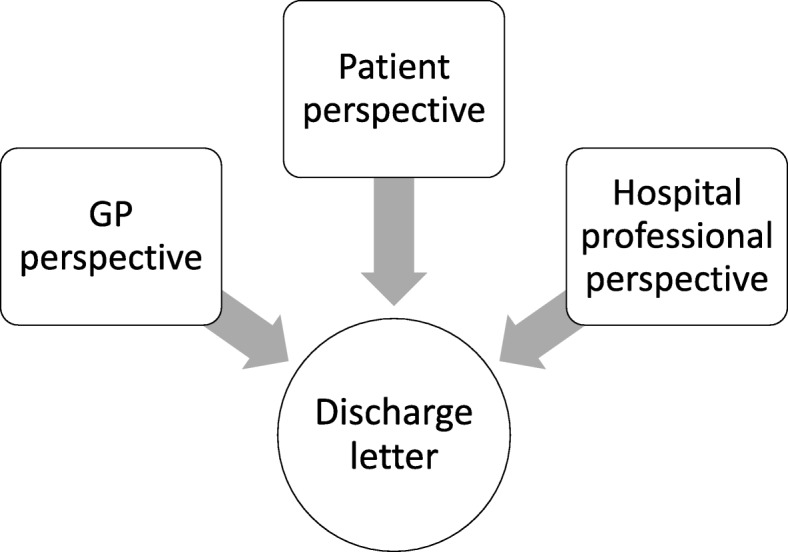
Discharge communication quartet

The study has a specific focus on patients receiving discharge letters and a secondary focus on discharge letter quality. The design allows each phase to capture different perspectives within the discharge communication process. This is being done through each participant’s perspective being “matched” to a specific discharge letter; alignment of perspectives for each letter then creates multiple viewpoint “cases”. We have termed these cases as “quartets” that map together the four elements, as illustrated in Fig. 1. Hence, discharge letters will be aligned with the perspective of the patient to whom the letter relates, the GP who received the letter, and the HP who wrote the letter. This process permits triangulation and comparison of different experiences within a single discharge event which allows for direct viewpoint comparisons and potential reconciliation of data disparities.

### Settings

The study involves purposive sampling [45, 46] at GP practices within the West Midlands (England, UK) to recruit a variety of GP, patient, and HP participants. The target was to recruit five large practices (> 10,000 patients), five medium practices (5000–10,000 patients), and five small practices (< 5000 patients) across a spread of urban/rural areas aiming for heterogeneity in terms of locality, affluence, size, patient demographics, and the hospitals from which they receive discharges. The hospitals that accounted for most discharge letters to these practices were eligible to participate, as were patients registered with these practices.

### Recruitment and data collection

**Fig. 2:**
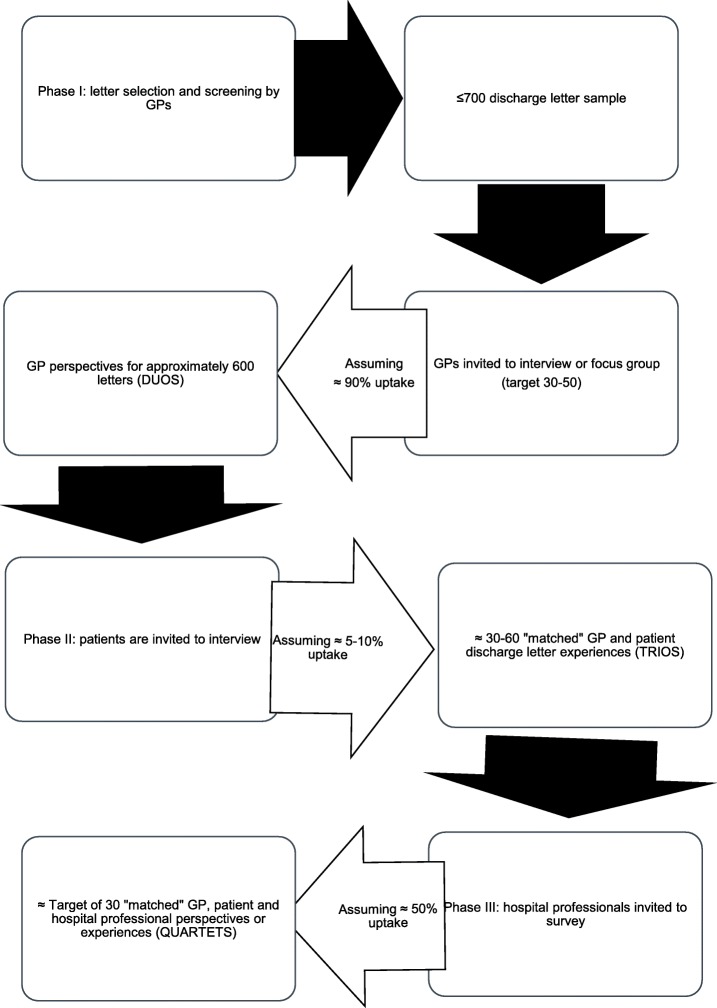
Recruitment and uptake assumptions for target of 30 “quartets”

The study aims to build 30 case “quartets”. Marshall et al. [47] report the average number of interviews for a qualitative study is 24 and that 15–30 interviews are generally recommended for data saturation to be achieved. Therefore, 30 quartets was considered to be an adequate sample size to produce findings that reflect the views of the groups included as participants. The recruitment assumptions to meet this aim are displayed in Fig. 2. They were informed by existing studies, patient and clinician involvement work, and expertise of the research team.

#### Phase I discharge letter sample selection and GP perspectives

Phase I opened in August 2017 and comprised two main parts: letter selection by GPs and GP perspectives through interviews and focus groups. GP practices were initially invited to take part through invitations circulated via the local primary care research network team (CRN), collaborating Clinical Commissioning Groups (CCGs) and Warwick Medical School links with practices. The study was also advertised through local primary care newsletters and at GP practice training events. Practices that expressed interest in participation were provided a study protocol and participation information sheet. Where practices requested more information before committing to participation, a site visit took place with a member of the research team or research network facilitator.

The study aimed to recruit 30–50 GPs across 15 practices, with a target of 2–3 GPs per practice. Participating GPs were asked to screen and select discharge letters as part of their routine practice review of letters in line with the study inclusion and exclusion criteria (see Table 3). It was not possible, within review scope, to include vulnerable patients (i.e. children/patients who lack capacity to give informed consent), those with particularly specialised communicative needs (i.e. unable to take part in English) or where participation may have a higher perceived risk of harm (e.g. psychiatric discharge). The needs of the excluded groups may be arguably more complex and variable within and between groups and warrant research in their own right. GPs were advised that letter selection determined the patients and HPs to be invited into phases II and III respectively and so inclusion and exclusion criteria applied to all phases. In addition, opt-out posters were provided for each participating practice to display to allow patients an opportunity to opt out of the study prior to letter selection, should they wish to do so.

**Table 3:** Study inclusion and exclusion criteria^a^

Inclusion criteria	• NHS adult (18+ years) patients discharged from a hospital following an episode of inpatient or outpatient care.
	• Patient registered with the participating GP practice.
	• Patient treated at and discharged from a hospital within Warwickshire, Coventry, Rugby, Herefordshire or Worcestershire.
	• Cases where written discharge communication has been sent to the patient’s GP.
Exclusion criteria	• Age < 18 years.
	• Patients who lack capacity to give informed consent to participate in the study (e.g. Alzheimer’s) or are deemed by the GP to be unsuitable for participation (e.g. end of life).
	• Patients discharged to providers or units other than their GP (e.g. discharge from hospital to a rehab unit).
	• Discharge communication from mental health services.
	• Communication about individuals who are considered unable to participate in an interview or focus group or survey conducted in English.
	• **Those who do not wish to participate.**
	• **Those who have expressed a general wish not to participate in research.**

^a^All criteria apply to phase I letters and phase II for patients with only those in bold applying to GPs in phase I and hospital professionals in phase III

In order to build the desired letter sample of 700 discharge letters, GPs had a target selection size of 14-24 letters each. Letters were sampled according to purposive sampling [48]; GPs were encouraged to choose letters for the study which they assessed to be “successful” or “unsuccessful” examples of discharge letters. There were no set criteria for letter categorisation as the selection was intended to be based on each participating GP’s interpretation of what makes successful or unsuccessful discharge letter. A selection template was completed by the GP for each sampled letter to record study ID code for the letter, the success grading (binary “successful”/“unsuccessful”), and comments on their reasons for their selection and categorisation. Comments, as with the categorisations, were entirely open; there were no guidelines or lists of reasons. Following letter selection, GP practice staff redacted the letters of patient identifiable information before transferral of the sample to the research team.

The GPs involved in letter selection were invited to take part in an interview or focus group with KW; these could take place face to face or over the telephone. Written consent was required for participation. The interviews and focus groups were “narrative” [49, 50] in style with a single opening question around GP experiences of discharge communication (see Additional file 2 for GP interview and focus group guide). The benefit of the narrative interview-style is that it was participant-led rather than researcher-led and hence there was potential for information to be revealed that was otherwise not anticipated or questioned [50]. GPs were encouraged to have sight of a copy of their letter sample and selection template to facilitate both discussion of their views on discharge letter content and patients receiving letters generally and in relation to the specific letters they selected for the study sample.

#### Phase II patient viewpoints

Phase II opened in October 2017. Patients associated with each of the discharge letters selected in phase I were sent by their practice an invitation pack for interview. The pack contained an invitation letter, patient information sheet, and copy of the consent form. It covered how and why the patient had been selected, the purpose of the study, risks and benefits of participation, research team details, and instructions of how to participate.

The invitation pack explained about the anonymised discharge letter sample and that one of the sampled letters related to recent contact that the patient had with the hospital. It explained that, if they wished, patients could withdraw their letter from the sample. It was also explained that the identity of the patient would only become known to the research team if the patient made contact with the team. Patients could contact the research team or their practice with any questions and to arrange an interview with KW at their GP practice or home. If they chose to participate, they would have the option to contact the research team to enquire about viewing the discharge letter at interview and that this would be enabled if their GP was in agreement. For those who had seen their letter previously, they were encouraged to bring or have this available at the interview. This design of letter availability for interviews, where possible, reduced recall bias. The target timeline for interviewing patients from their date of hospital discharge was 4–6 weeks.

Prior to commencement of interview, the reasons for the research and right to withdraw were reiterated. Written consent was required for all patient interviews in the presence of the interviewer (KW). As far as was possible, the setting and length of interviews was accommodated to participants’ preferences. The running time was flexible although expected to last between 30 min and an hour.

The patient interviews were “semi-structured” [50] with eight open questions based on their experiences of discharge communication, their views on their recent letter (if applicable), as well as their preferences for receiving discharge letters and how they feel discharge communication can be improved (see Additional file 3 for patient interview guide). Towards the end of the interview, the participant was invited to add any further thoughts, reflections or comments. Patients were thanked for participation and offered a £20 multi-site high street voucher as a token of gratitude, with the options also to decline or donate the voucher. In addition, any out of pocket expenses incurred could be reimbursed if the patient notified the research team. After interviews, patients were provided the opportunity to have a copy of their signed consent form and given a post-interview support sheet; this signposted them to different services for any queries or concerns (e.g. complaints) in regard to their discharge experience.

#### Phase III Hospital professional experiences

Phase III opened in May 2018. HPs who wrote or signed the discharge letters selected by the GPs in phase I were invited to take part in a survey. Invitation packs for the survey were sent to eligible individuals in external post by the research team or delivered to the hospital for internal distribution. Invitation packs contained an invitation letter explaining how and why they have been selected, a participant information sheet with further details of the study, a survey, and a redacted copy of the letter they wrote/signed that had been included in the sample.

The survey questionnaire comprised 15 questions covering the HP’s assessment of their letter, their current practices and views on patients receiving or not receiving letters, and how they think discharge communication can be improved. The closed question formats involved discrete ‘semantic differential’ scales [51, 52] and closed multiple choice check boxes. There was also a single open question at the end of the survey, so as not to deter participation [51, 53], that invited HPs to provide reasons for their answers or add any other comments. In addition, there were demographic and administrative questions at the start and end of the survey. The survey length was intended to be brief, with an anticipated completion time of 5–15 min dependent on the extent to which the participant chose to provide a free text response.

Invited HPs were given up to 6 weeks’ to respond to the survey invitation; this was deemed sufficient to balance time to allow consideration of participation whilst ensuring the recruitment period would be of feasible duration. Up to three reminders could be sent directly via the research team (e.g. email) or internally within the hospital (e.g. Research and Development department communications).

### Data analysis

The study design is mixed methods and will combine approaches and analytical techniques from the fields of Applied Linguistics and Health Sciences. Each phase involves application of different techniques for analysis; these are outlined below. The COREQ checklist by Tong et al. [54] for qualitative reporting will be used to structure analyses and reporting of findings.

#### Corpus linguistics

Analysis of the interview and focus group data from phases I and II will involve linguistic methods, namely, corpus-driven [55, 56] techniques. *Corpus linguistics* (CL) is the study of language through corpora [57] (plural of *corpus*), which are electronic, machine readable ‘collections of texts’ [58]. CL focuses on analysing patterns of co-occurrence and meanings in data. Corpus processing can reveal language patterns and commonalities as well as rare cases; neither of which are likely to be reliably available through manual searching or intuition alone [59, 60]. CL is particularly useful for the current study as it allows rapid scrutiny of a large body of qualitative data through both quantitative and qualitative analyses [61], and so is particularly suited to the analysing the GP and patient data.

Initially, all interviews and focus group recorded data will be transcribed by KW using standard orthographic transcription [62]; any identifiable features will be removed and replaced with generic terms e.g. [NAME]. Self-transcribing aims to ensure uniformity across transcripts and increase data familiarity [59]. Next, copies of formatted transcripts will be imported into *Antconc* [63], a specialist linguistic software or *concordancer* [57], to build two corpora; one for GP data and one for patient data.

In line with a predominantly corpus-driven [64] approach and following previous corpus linguistics health-focussed papers [65, 66], quantitative techniques in the form of *keyword* lists [57] will be used as a point of departure for identifying salient linguistic features and “patterns” [67, 68]. The BNC Spoken (2014) [62] will be used as a reference corpus for generation of key words. The statistical calculation for keywords will be log-likelihood (5% level; *p* < 0.05). Thereafter, qualitative techniques, informed by the quantitative findings, to investigate and examine *collocations* [69] and *concordance lines* [57] will be undertaken; this will allow more in depth exploration of quantitative findings [57, 70]. Again, the statistical calculation for generating collocates will be log-likelihood (*p* < 0.05).

The corpora will also be statistically mined for measures of dispersion [71, 72] of salient linguistic items and patterns. Hence, we will analyse the corpora through triangulation of different CL techniques to increase analytical robustness and validity of findings [73]. Overall, interview and focus group data analysis will be corpus-driven using qualitative and quantitative techniques in order to address RQs1–4.

#### Content analysis

The letter sample from phase I will be interrogated and assessed using content analysis [74]. Letters will be coded in respect of presence or absence of specific content features (e.g. diagnosis, medication); feature categories will be guided by *The Royal College of Physicians* [75] standards for content and structure of records. Features coded between the successful/unsuccessful GP letter groupings will then be quantitatively compared using inferential non-parametric statistics (Chi-square, *p* < 0.05) in order to test hypotheses for differences between discharge letter content features of the two groups of letters. It is anticipated that this may reveal insights into what makes a successful or unsuccessful letter according to GPs; these insights have direct relevance to RQ4 and may shed light upon how discharge letters can be improved. Additionally, any GP comments included on the study GP letter selection template will be analysed using corpus linguistics employing the techniques described above. Using CL methods for GP comments permits triangulation with content analysis findings for the purposes of confirmation and explanation of findings, or otherwise.

#### Statistical analyses

Across all phases, demographic information, where provided, will be analysed descriptively. Inferential and descriptive statistics will be used to describe sample representativeness as well as for hypothesis-testing, where applicable, to ascertain whether there are differences of viewpoints on discharge communication between demographic and phase groups.

HP survey results will be explored using descriptive and inferential statistics. Survey data will be analysed with appropriate statistics and most likely presented with frequency tables, percentages, means, range, median, IQR, and, where appropriate, standard deviation or skewness and kurtosis scores [51]. The free text data will be narratively overviewed; CL analysis may be undertaken if there are a large number of responses. Additionally, where possible, inferential statistical analyses or hypothesis-testing [76] statistics will be conducted. Independent variables will take the form of any disclosed sociodemographic (e.g. age) or administrative information (e.g. hospital role).

#### Integration of analyses

Findings from across phases will be integrated in a secondary-level data analysis through use of meta-matrices [77] to allow synthesis of qualitative and quantitative findings across phases. Additionally, individual perspectives across phases will be matched to specific discharge letters within the sample to build “quartet” cases (target = 30 with 90 unique perspectives from phases). The juxtaposition of perspectives will be used to highlight convergence, divergence, and trends between different groups. This will aim to provide insights particularly around best practice of patients receiving letters, and make practicable recommendations for how discharge communication in terms of patients receiving letters (RQ1–3) and content (RQ4) may be improved. In addition, the programme theory from the realist review will be further developed based on primary data phase analysis in order to generate a resultant theory for when patients receiving and not receiving discharge letters does and does not “work”.

### Patient and public involvement

The project’s overall methodological approach involves patient and public involvement (PPI), clinician, and policy maker involvement in a process of collaboration [31]. This was undertaken in the design stage and is also planned for interpretation of results and consideration of the research finding implications for practice. The objective is that participants and stakeholders who collaborated in the study will be contacted toward the completion of data analysis; they will be provided a results summary and invited to assist interpretation of results. This stage of involvement will mirror involvement methods in the design phase and thus may take a variety forms depending on preference of those involved and feasibility limitations. These forms may include but are not limited to: panel and group discussions (e.g. discussion of findings with local PPGs), telephone and electronic feedback (e.g. communications with service users who provided email or phone contact details to hear about results in phase II), and presentations (e.g. research team present at meetings with collaborating CCGs). This involvement work is intended to increase the relevance and impact of the research findings.

## Discussion

The study is particularly timely given the recently published initiative ‘Please write to me’ [5], by the Academy of Medical Royal Colleges in the UK, which focuses on patients receiving outpatient letters. Our research focuses on improvement of discharge communication in relation to patients receiving inpatient and outpatient letters (RQs1–3) and content of written discharge letters (RQ4). The study is an innovative exploratory design which triangulates the perspectives of patients, HPs, and GPs in relation to specific discharge communication events. The study’s findings have the potential to inform recommendations for improving discharge communication.

The study is strengthened by the number and diversity of participating sites. The research aims to build a sample of 700 letters and recruit a minimum of 30 GPs, 30 patients and 30 HPs in order to capture 90 unique experiences of discharge communication and, through integration of findings, build 30 matched case “quartets”. The sampling strategies for both letters and participants are designed to encompass heterogeneity of discharges (e.g. type of admission, age of patient, discharge speciality …) in an attempt to build a somewhat representative sample of discharge letters and discharge experiences. Moreover, the binary differentiations for sampling between GP-assessed “successful” and “unsuccessful” letters seeks to increase sample diversity and relevance of findings through purposive sampling. Nonetheless, recruitment is limited to a single region (West Midlands, UK) and the study exclusion and inclusion criteria imposes some restrictions; children (< 18 years), persons unable to take part in English, discharges relating solely to mental health, and cases deemed by GP to lack capacity to give informed consent or be unsuitable for the study are excluded. These exclusions limit the applicability and generalisability of findings and therefore the sample cannot be considered entirely representative of UK written discharge communications.

The study design aims to reflect the research team values of equality and diversity. Participants may have found taking part in the study burdensome (e.g. financially, physically…) if they were required to travel to participate. This could have raised potential inequity issues in terms of access to the study. To tackle these issues, participation for each phase group is tailored to accommodate preferences of the participants as far as was possible. GP interviews and focus groups could be face to face or over the telephone. Patient interviews could take place at their GP practice or home. HP surveys could be completed in hard copy or electronically and returned via either mode. Study packs encouraged those who felt burdened or had concerns around study accessibility to contact the research team to discuss their individual needs so that, within feasibility limitations, these could hopefully be addressed.

The planned data analysis has strengths and limitations. Analysis and comparisons of aligned multiple perspectives through “quartets” should provide new insights into some of the previous reported discrepancies on the impacts of patients receiving letters. It is expected that for some discharge letters, it will not be possible to form a complete quartet. Nonetheless, participant viewpoints and letter analysis will still provide data and valuable perspectives relevant to the study research questions. There is inherent subjectivity in GPs’ selection of the discharge letter sample. We specifically wanted to understand from the perspective of GPs, what constitutes successful communication and what is seen as being unsuccessful. Qualitative data analysis can be subjective and interpretation of qualitative data may be limited by the researcher; individual identities and attitudes inherently impact upon data interpretations [78]. Therefore, “reflexivity” will be practised throughout the research to account for this subjectivity and reduce but not eradicate bias [78, 79]. The quantitative methods involved in the corpus-driven analysis of qualitative data augments accountability and replicability of the findings in order to increase validity and reliability and satisfy falsifiability standards [64]. Nevertheless, quantitative analysis is limited by the study sample; across phases, due to small sample sizes and predominantly categorical variables, parametric testing is unlikely to be possible.

### Ethics

Ethics approval was granted in July 2017 by the National Health Service Health Research Authority (IRAS ID: 219871, REC reference: 17/WM/0170). A summary of the ethical issues considered and risks of this study as well as actions to minimise these issues is in Additional file 4.

### Dissemination and outputs

Results will in part be disseminated through the patient and public involvement planned throughout and toward the end of the study. Moreover, the aim is for patient and public involvement input in the final stages of finding interpretations to inform and support dissemination plans. It is anticipated that findings will be disseminated to a range of audiences (i.e. policy-makers, clinicians…) as peer-reviewed journals, newsletters, presentations, and conferences. Reports in plain English will be prepared for participants, participating sites, local and collaborating policy-makers and commissioners, and shared with any other interested parties. This research also forms part of a PhD thesis. It is anticipated that the results will aid informing guidelines on discharge communication. Recommendations for improving discharge letters will be made.

## Supplementary information

**Additional file 1.** List of included documents in scoping literature review.

**Additional file 2.** GP interview and focus group guide.

**Additional file 3.** Patient interview guide.

**Additional file 4.** Table summary of ethical issues and research team responses.

## Data Availability

The datasets generated during the current study are not publicly available due to the sensitive and identifiable nature of the data. Despite names and other identifiers being removed, the in depth nature of the interviews themselves may mean that participants can be identified. Quotes will be used in results publications and further quotations are available from the corresponding author on reasonable request.

## Abbreviations

CCG: Clinical Commissioning Group
CRN: Clinical Research Network
GP: General Practitioner (UK family or primary care physician)
HP: Hospital professional
HRA: Health Research Authority
IQR: Interquartile range
IRAS: Integrated Research Application System
NHS: UK National Health Service
PPI: Patient and public involvement
REC: Research Ethics Committee
UK: United Kingdom
