# Supplementary material for: The Discharge Communication Study: research protocol for a mixed methods study to investigate and triangulate discharge communication experiences of patients, GPs, and hospital professionals, alongside a corresponding discharge letter sample
Source: BMC Health Serv Res. 2019 Nov 11;19:825. doi: 10.1186/s12913-019-4612-1 (PMC6849198; doi:10.1186/s12913-019-4612-1)
Supplement: Supplementary file 1 — Additional file 1. List of included documents in scoping literature review. [file 12913_2019_4612_MOESM1_ESM.docx]

*List of included documents in scoping literature review*

1.Spinewine A., Claeys C., Foulon V., Chevalier P. Approaches for improving continuity of care in medication management: a systematic review. *Int J Qual Health Care*. 2013;25(4):403-17.

2.Motamedi S.M., Posadas-Calleja J., Straus S., Bates D.W., Lorenzetti D.L., Baylis B., Gilmour J., Kimpton S., Ghali W.A. The efficacy of computer-enabled discharge communication interventions: a systematic review. *BMJ Qual Saf*. 2011;20(5):403-15.

3.Closs S. ‘Discharge Communications between Hospital and Community Health Care Staff: A Selective Review’. *Health and Social Care in the Community*. 1996;5(3):181-97.

4.Shepperd S., Parkes J., McClaren J., Phillips C. Discharge planning from hospital to home. *Cochrane Database Syst Rev*. 2004 (1).

5.Kripalani S., Jackson A.T., Schnipper J.L., Coleman E.A. Promoting effective transitions of care at hospital discharge: a review of key issues for hospitalists. *J Hosp Med*. 2007;2(5):314-23.

6.Mistiaen P., Francke A.L., Poot E. Interventions aimed at reducing problems in adult patients discharged from hospital to home: a systematic meta-review. *BMC Health Serv Res*. 2007;7:47-.

7.Dunn A.S., Markoff B. Physician-physician communication: what's the hang-up? *J Gen Intern Med*. 2009;24(3):437-9.

8.Shepperd S., McClaran J., Phillips C.O., Lannin N.A., Clemson L.M., McCluskey A., Cameron I.D., Barras S.L. Discharge planning from hospital to home. *Cochrane Database Syst Rev*. 2010 (1).

9.Hesselink G., Schoonhoven L., Barach P., Spijker A., Gademan P., Kalkman C., Liefers J., Vernooij-Dassen M., Wollersheim H. Improving patient handovers from hospital to primary care: a systematic review. *Ann Intern Med*. 2012;157(6):417-28.

10.Alberti T.L., Nannini A. Patient comprehension of discharge instructions from the emergency department: a literature review. *J Am Assoc Nurse Pract*. 2013;25(4):186-94.

11.Wimsett J., Harper A., Jones P. Review article: Components of a good quality discharge summary: a systematic review. *Emergency Medicine Australasia*. 2014;26(5):430-8.

12.Mills P.R., Weidmann A.E., Stewart D. Hospital discharge information communication and prescribing errors: a narrative literature overview. *European Journal of Hospital Pharmacy*. 2015.

13.Unnewehr M., Schaaf B., Marev R., Fitch J., Friederichs H. Optimizing the quality of hospital discharge summaries--a systematic review and practical tools. *Postgrad Med*. 2015;127(6):630-9.

14.Sandler D.A., Mitchell J.R. Interim discharge summaries: how are they best delivered to general practitioners? *British Medical Journal Clinical Research Ed*. 1987;295(6612):1523-5.

15.Penney T.M. Dictate a discharge summary. *Bmj*. 1989;298(6680):1084-5.

16.King M.H., Barber S.G. Towards better discharge summaries: brevity and structure. *West of England Medical Journal*. 1991;106(2):40-1, 55.

17.Colledge N.R., Smith R.G., Lewis S.J. The delivery of interim discharge summaries to general practitioners by the elderly. *Health Bulletin*. 1992;50(3):219-22.

18.Jorsh M.S., Palmer D.J. GPs' opinions on the use of an interim discharge summary for psychiatric inpatients. *British Journal of General Practice*. 1992;42(358):212.

19.Adams D.C., Bristol J.B., Poskitt K.R. Surgical discharge summaries: improving the record. *Annals of the Royal College of Surgeons of England*. 1993;75(2):96-9.

20.Barr F. ‘CQC Raises Discharge Concerns: The Discharge Summary Target’. *E-Health Insider*. 2010 http://www.digitalhealth.net/news/25571/.

21.Wilson S., Ruscoe W., Chapman M., Miller R. General practitioner-hospital communications: a review of discharge summaries. *J Qual Clin Pract*. 2001;21(4):104-8.

22.Belleli E., Naccarella L., Pirotta M. Communication at the interface between hospitals and primary care - a general practice audit of hospital discharge summaries. *Australian Family Physician*. 2013;42(12):886-90.

23.Li J.Y., Yong T.Y., Hakendorf P., Ben-Tovim D., Thompson C.H. Timeliness in discharge summary dissemination is associated with patients' clinical outcomes. *J Eval Clin Pract*. 2013;19(1):76-9.

24.van Walraven C., Seth R., Laupacis A. Dissemination of discharge summaries. Not reaching follow-up physicians. *Canadian Family Physician*. 2002;48:737-42.

25.Okoniewska B.M., Santana M.J., Holroyd-Leduc J., Flemons W., O'Beirne M., White D., Clement F., Forster A., Ghali W.A. The Seamless Transfer-of-Care Protocol: a randomized controlled trial assessing the efficacy of an electronic transfer-of-care communication tool. *BMC Health Serv Res*. 2012;12:414.

26.Polyzotis P.A., Suskin N., Unsworth K., Reid R.D., Jamnik V., Parsons C., Grace S.L. Primary care provider receipt of cardiac rehabilitation discharge summaries: are they getting what they want to promote long-term risk reduction? *Circulation Cardiovascular Quality & Outcomes*. 2013;6(1):83-9.

27.Shen M.W., Hershey D., Bergert L., Mallory L., Fisher E.S., Cooperberg D. Pediatric hospitalists collaborate to improve timeliness of discharge communication. *Hospital Pediatrics*. 2013;3(3):258-65.

28.Salim Al-Damluji M., Dzara K., Hodshon B., Punnanithinont N., Krumholz H.M., Chaudhry S.I., Horwitz L.I. Association of discharge summary quality with readmission risk for patients hospitalized with heart failure exacerbation. *Circulation Cardiovascular Quality & Outcomes*. 2015;8(1):109-11.

29.Curran P., Gilmore, D. & Beringer, T. . ‘Communication of Discharge Information for Elderly Patients in Hospital’. *Ulster Medical Journal*. 1992;61(1):Pp. 56-8.

30.Archbold R.A., Laji K., Suliman A., Ranjadayalan K., Hemingway H., Timmis A.D. Evaluation of a computer-generated discharge summary for patients with acute coronary syndromes. *British Journal of General Practice*. 1998;48(429):1163-4.

31.Branger P.J., van der Wouden J.C., Schudel B.R., Verboog E., Duisterhout J.S., van der Lei J., van Bemmel J.H. Electronic communication between providers of primary and secondary care. *Bmj*. 1992;305(6861):1068-70.

32.van Walraven C., Weinberg A.L. Quality assessment of a discharge summary system. *CMAJ Canadian Medical Association Journal*. 1995;152(9):1437-42.

33.Crosswhite R., Beckham S.H., Gray P., Hawkins P.R., Hughes J. Using a multidisciplinary automated discharge summary process to improve information management across the system. *American Journal of Managed Care*. 1997;3(3):473-9.

34.van Walraven C., Laupacis A., Seth R., Wells G. Dictated versus database-generated discharge summaries: a randomized clinical trial. *CMAJ Canadian Medical Association Journal*. 1999;160(3):319-26.

35.Barr R., Chin K.Y., Yeong K. Improving transmission rates of electronic discharge summaries to GPs. *BMJ Qual Improv Rep*. 2013;2(1).

36.Moran W.P., Davis K.S., Moran T.J., Newman R., Mauldin P.D. Where are my patients? It is time to automate notification of hospital use to primary care practices. *Southern Medical Journal*. 2012;105(1):18-23.

37.Hunchak C., Tannenbaum D., Roberts M., Shah T., Tisma P., Ovens H., Borgundvaag B. Closing the circle of care: implementation of a web-based communication tool to improve emergency department discharge communication with family physicians. *Cjem*. 2015;17(2):123-30.

38.Stetson P.D., Keselman A., Rappaport D., Van Vleck T., Cooper M., Boyer A., Hripcsak G. Electronic discharge summaries. *AMIA* 2005;Annual Symposium Proceedings/AMIA Symposium.:1121.

39.Sevick L.K., Santana M.J., Ghali W.A., Clement F. Prospective economic evaluation of an electronic discharge communication tool: analysis of a randomised controlled trial. *BMJ Open*. 2017;7(12).

40.Schabetsberger T., Ammenwerth E., Andreatta S., Gratl G., Haux R., Lechleitner G., Schindetwig K., Stark C., Vogl R., Wilhelmy I., Wozak F. From a paper-based transmission of discharge summaries to electronic communication in health care regions. *Int J Med Inform*. 2006;75(3-4):209-15.

41.Sheu L., Fung K., Mourad M., Ranji S., Wu E. We need to talk: Primary care provider communication at discharge in the era of a shared electronic medical record. *Journal of Hospital Medicine (Online)*. 2015;10(5):307-10.

42.Abbas M., Ward T., Peivandi M., McKenzie E., Kujawska-Debiec K., Hills A. Quality of psychiatric discharge summaries: a service evaluation following the introduction of an electronic discharge summary system. *Irish Journal of Psychological Medicine*. 2015;32(4):327-30.

43.Shannon D. Effective physician-to-physician communication: an essential ingredient for care coordination. *Physician Exec*. 2012;38(1):16-21.

44.Jansen J.O., Grant I.C. Communication with general practitioners after accident and emergency attendance: computer generated letters are often deficient. *Emergency Medicine Journal*. 2003;20(3):256-7.

45.Stainkey L., Pain T., McNichol M., Hack J., Roberts L. Matched comparison of GP and consultant rating of electronic discharge summaries. *Health Information Management Journal*. 2010;39(3):7-15.

46.Chen Y., Brennan N., Magrabi F. Is email an effective method for hospital discharge communication? A randomized controlled trial to examine delivery of computer-generated discharge summaries by email, fax, post and patient hand delivery. *Int J Med Inform*. 2010;79(3):167-72.

47.Lane N., Bragg M.J. From emergency department to general practitioner: evaluating emergency department communication and service to general practitioners. *Emergency Medicine Australasia*. 2007;19(4):346-52.

48.Tay L. Cochrane Review Brief: Email for Clinical Communication Between Healthcare Professionals. *Online J Issues Nurs*. 2013;18(3):11.

49.NHS England. ‘Transfer of Care – eDischarge’. 2015 https://www.england.nhs.uk/digitaltechnology/info-revolution/interoperability/transfer-of-care-edischarge/. .

50.van Walraven C., Rokosh E. What is necessary for high-quality discharge summaries? *Am J Med Qual*. 1999;14(4):160-9.

51.Solomon J.K., Maxwell R.B., Hopkins A.P. Content of a discharge summary from a medical ward: views of general practitioners and hospital doctors. *Journal of the Royal College of Physicians of London*. 1995;29(4):307-10.

52.Castleden W.M., Stacey M.C., Norman P.E., Lawrence-Brown M.M., Brooks J.G. General practitioners' attitudes to computer-generated surgical discharge letters. Members of the Department of General Surgery, Fremantle Hospital. *Med J Aust*. 1992;157(6):380-2.

53.Lees L. Improving the quality of patient discharge from emergency settings. *British Journal of Nursing*. 2004;13(7):412-6,8,21.

54.Groene R.O., Orrego C., Sunol R., Barach P., Groene O. "It's like two worlds apart": an analysis of vulnerable patient handover practices at discharge from hospital. *BMJ Qual Saf*. 2012;21 Suppl 1:i67-75.

55.Hammad E.A., Wright D.J., Walton C., Nunney I., Bhattacharya D. Adherence to UK national guidance for discharge information: an audit in primary care. *British Journal of Clinical Pharmacology*. 2014;78(6):1453-64.

56.Mamo J.P. Electronic discharge summaries--are they being done and do they have the required information? *Ir Med J*. 2014;107(3):88-90.

57.Cresswell A., Hart M., Suchanek O., Young T., Leaver L., Hibbs S. Mind the gap: Improving discharge communication between secondary and primary care. *BMJ Qual Improv Rep*. 2015;4(1).

58.Pillai A., Thomas S., Garg M. The electronic immediate discharge document: experience from the South West of Scotland. *Journal of Innovation in Health Informatics*. 2004;12(2):67-73.

59.Stein R., Neufeld D., Shwartz I., Erez I., Haas I., Magen A., Glassberg E., Shmulevsky P., Paran H. Assessment of surgical discharge summaries and evaluation of a new quality improvement model. *Israel Medical Association Journal: Imaj*. 2014;16(11):714-7.

60.Santana M.J., Holroyd-Leduc J., Flemons W.W., O'Beirne M., White D., Clayden N., Forster A.J., Ghali W.A. The seamless transfer of care: a pilot study assessing the usability of an electronic transfer of care communication tool. *American Journal of Medical Quality*. 2014;29(6):476-83.

61.Halasyamani L., Kripalani S., Coleman E., Schnipper J., van Walraven C., Nagamine J., Torcson P., Bookwalter T., Budnitz T., Manning D. Transition of care for hospitalized elderly patients--development of a discharge checklist for hospitalists. *J Hosp Med*. 2006;1(6):354-60.

62.Moore C., McGinn T., Halm E. Tying up loose ends: discharging patients with unresolved medical issues. *Archives of Internal Medicine*. 2007;167(12):1305-11.

63.Borowitz S.M., Waggoner-Fountain L.A., Bass E.J., Sledd R.M. Adequacy of information transferred at resident sign-out (in-hospital handover of care): a prospective survey. *Qual Saf Health Care*. 2008;17(1):6-10.

64.Kind A.J.H., Smith M.A. Advances in Patient Safety: Documentation of Mandated Discharge Summary Components in Transitions from Acute to Subacute Care. In: Henriksen K., Battles J.B., Keyes M.A., Grady M.L., editors. *Advances in Patient Safety: New Directions and Alternative Approaches (Vol 2: Culture and Redesign)*. Rockville (MD): Agency for Healthcare Research and Quality (US); 2008.

65.Were M.C., Li X., Kesterson J., Cadwallader J., Asirwa C., Khan B., Rosenman M.B. Adequacy of hospital discharge summaries in documenting tests with pending results and outpatient follow-up providers. *Journal of General Internal Medicine*. 2009;24(9):1002-6.

66.Walz S.E., Smith M., Cox E., Sattin J., Kind A.J. Pending laboratory tests and the hospital discharge summary in patients discharged to sub-acute care. *Journal of General Internal Medicine*. 2011;26(4):393-8.

67.Kind A.J., Thorpe C.T., Sattin J.A., Walz S.E., Smith M.A. Provider characteristics, clinical-work processes and their relationship to discharge summary quality for sub-acute care patients. *Journal of General Internal Medicine*. 2012;27(1):78-84.

68.Flink M., Bergenbrant Glas S., Airosa F., Ohlen G., Barach P., Hansagi H., Brommels M., Olsson M. Patient-centered handovers between hospital and primary health care: an assessment of medical records. *Int J Med Inform*. 2015;84(5):355-62.

69.Kantor M.A., Evans K.H., Shieh L. Pending studies at hospital discharge: a pre-post analysis of an electronic medical record tool to improve communication at hospital discharge. *Journal of General Internal Medicine*. 2015;30(3):312-8.

70.Smith V.C., Dukhovny D., Zupancic J.A., Gates H.B., Pursley D.M. Neonatal intensive care unit discharge preparedness: primary care implications. *Clinical Pediatrics*. 2012;51(5):454-61.

71.Peacock J.J. Discharge summary for medically complex infants transitioning to primary care. *Neonatal Network - Journal of Neonatal Nursing*. 2014;33(4):204-7.

72.Tattersall M.H., Butow P.N., Brown J.E., Thompson J.F. Improving doctors' letters. *Med J Aust*. 2002;177(9):516-20.

73.Alderton M., Callen J. Are general practitioners satisfied with electronic discharge summaries? *Health Information Management Journal*. 2007;36(1):7-12.

74.Singh G., Harvey R., Dyne A., Said A., Scott I. Hospital discharge summary scorecard: a quality improvement tool used in a tertiary hospital general medicine service. *Internal medicine journal*. 2015;45(12):1302-5.

75.Royal College of Physicians. Standards for the clinical structure and content of patient records. 2013 https://www.rcplondon.ac.uk/projects/outputs/standards-clinical-structure-and-content-patient-records.

76.The Joint Commission. ‘Joint Commission on Accreditation of Healthcare Organizations.’ *National Patient Safety Goals Hospital Program*. 2008 www.jointcommission.org/PatientSafety/NationalPatientSafetyGoals/08_hap_npsgs.htm.

77.Sandler D.A., Heaton C., Garner S.T., Mitchell J.R. Patients' and general practitioners' satisfaction with information given on discharge from hospital: audit of a new information card. *Bmj*. 1989;299(6714):1511-3.

78.Paterson J.M., Allega R.L. Improving communication between hospital and community physicians. Feasibility study of a handwritten, faxed hospital discharge summary. Discharge Summary Study Group. *Canadian Family Physician*. 1999;45:2893-9.

79.Makela P., Haynes C., Holt K., Kar A. Written medical discharge communication from an acute stroke service: a project to improve content through development of a structured stroke-specific template. *BMJ Qual Improv Rep*. 2013;2(1).

80.Leyenaar J.K., Bergert L., Mallory L.A., Engel R., Rassbach C., Shen M., Woehrlen T., Cooperberg D., Coghlin D. Pediatric primary care providers' perspectives regarding hospital discharge communication: a mixed methods analysis. *Academic Pediatrics*. 2015;15(1):61-8.

81.Parks T., Kingham E., McEwen D., Cooper S. The preference of general practitioners for structured outpatient clinic letters. *Clin Med (Lond)*. 2011;11(2):205-6.

82.Lockman K.A., Lee W.H., Sinha R., Teoh W.L., Bickler C., Dummer S., Veiraiah A. Effective acute care handover to GP: optimising the structure to improve discharge documentation. *Acute Med*. 2018;17(2):68-76.

83.van Walraven C., Duke S.M., Weinberg A.L., Wells P.S. Standardized or narrative discharge summaries. Which do family physicians prefer? *Canadian Family Physician*. 1998;44:62-9.

84.Tsilimingras D., Bates D.W. Addressing postdischarge adverse events: a neglected area. *The Joint Commission Journal on Quality and Patient Safety*. 2008;34(2):85-97.

85.Rao P., Andrei A., Fried A., Gonzalez D., Shine D. Assessing quality and efficiency of discharge summaries. *American Journal of Medical Quality*. 2005;20(6):337-43.

86.Maslove D.M., Leiter R.E., Griesman J., Arnott C., Mourad O., Chow C.M., Bell C.M. Electronic versus dictated hospital discharge summaries: a randomized controlled trial. *Journal of General Internal Medicine*. 2009;24(9):995-1001.

87.Axon R.N., Penney F.T., Kyle T.R., Zapka J., Marsden J., Zhao Y., Mauldin P.D., Moran W.P. A hospital discharge summary quality improvement program featuring individual and team-based feedback and academic detailing. *American Journal of the Medical Sciences*. 2014;347(6):472-7.

88.Sorita A., Robelia P.M., Kattel S.B., McCoy C.P., Keller A.S., Almasri J., Murad M.H., Newman J.S., Kashiwagi D.T. The Ideal Hospital Discharge Summary: A Survey of U.S. Physicians. *Journal of patient safety*. 2017.

89.Afilalo M., Lang E., Léger R., Xue X., Colacone A., Soucy N., Vandal A., Boivin J.-F., Unger B. Impact of a standardized communication system on continuity of care between family physicians and the emergency department. *Canadian Journal of Emergency Medicine*. 2007;9(2):79-86.

90.Callen J., McIntosh J., Li J. Accuracy of medication documentation in hospital discharge summaries: A retrospective analysis of medication transcription errors in manual and electronic discharge summaries. *Int J Med Inform*. 2010;79(1):58-64.

91.Frain J.P., Frain A.E., Carr P.H. Experience of medical senior house officers in preparing discharge summaries. *Bmj*. 1996;312(7027):350.

92.Macaulay E.M., Cooper G.G., Engeset J., Naylor A.R. Prospective audit of discharge summary errors. *British Journal of Surgery*. 1996;83(6):788-90.

93.Legault K., Ostro J., Khalid Z., Wasi P., You J.J. Quality of discharge summaries prepared by first year internal medicine residents. *BMC Medical Education*. 2012;12(1):77.

94.Tan B., Mulo B., Skinner M. Transition from hospital to primary care: an audit of discharge summary - medication changes and follow-up expectations. *Internal medicine journal*. 2014;44(11):1124-7.

95.Yemm R., Bhattacharya D., Wright D., Poland F. What constitutes a high quality discharge summary? A comparison between the views of secondary and primary care doctors. *International Journal of Medical Education*. 2014;5:125-31.

96.Talwalkar J.S., Ouellette J.R., Alston S., Buller G.K., Cottrell D., Genese T., Vaezy A. A structured workshop to improve the quality of resident discharge summaries. *Journal of Graduate Medical Education*. 2012;4(1):87-91.

97.Myers J.S., Jaipaul C.K., Kogan J.R., Krekun S., Bellini L.M., Shea J.A. Are discharge summaries teachable? The effects of a discharge summary curriculum on the quality of discharge summaries in an internal medicine residency program. *Academic Medicine*. 2006;81(10 Suppl):S5-8.

98.Carnahan J.L., Fletcher K.E. Discharge Education for Residents: A Study of Trainee Preparedness for Hospital Discharge. *WMJ*. 2015;114(5):185-9.

99.Dinescu A., Fernandez H., Ross J.S., Karani R. Audit and feedback: an intervention to improve discharge summary completion. *Journal of Hospital Medicine (Online)*. 2011;6(1):28-32.

100.Russell P., Hewage U., Thompson C. Method for improving the quality of discharge summaries written by a general medical team. *Internal medicine journal*. 2014;44(3):298-301.

101.Record J.D., Niranjan-Azadi A., Christmas C., Hanyok L.A., Rand C.S., Hellmann D.B., Ziegelstein R.C. Telephone calls to patients after discharge from the hospital: an important part of transitions of care. *Med Educ Online*. 2015;20:26701.

102.Shaikh U., Slee C. Triple Duty: Integrating Graduate Medical Education With Maintenance of Board Certification to Improve Clinician Communication at Hospital Discharge. *Journal of Graduate Medical Education*. 2015;7(3):462-5.

103.Shivji F.S., Ramoutar D.N., Bailey C., Hunter J.B. Improving communication with primary care to ensure patient safety post-hospital discharge. *British Journal of Hospital Medicine*. 2015;76(1):46-9.

104.Yates N., Brazil V. Get the DRIFT for great discharge summaries. *Emergency Medicine Australasia*. 2015;27(3):273.

105.Key-Solle M., Paulk E., Bradford K., Skinner A.C., Lewis M.C., Shomaker K. Improving the quality of discharge communication with an educational intervention. *Pediatrics*. 2010;126(4):734-9.

106.Bischoff K., Goel A., Hollander H., Ranji S.R., Mourad M. The Housestaff Incentive Program: improving the timeliness and quality of discharge summaries by engaging residents in quality improvement. *BMJ Qual Saf*. 2013;22(9):768-74.

107.Tejedor-Sojo J., Creek T., Leong T. Impact of audit and feedback and pay-for-performance interventions on pediatric hospitalist discharge communication with primary care providers. *American Journal of Medical Quality*. 2015;30(2):149-55.

108.British Medical Journal. 'Planning a patient’s discharge from hospital’ *Bmj*. 2008;337(a2694. ).

109.Siders A.M., Peterson M. Increasing patient satisfaction and nursing productivity through implementation of an automated nursing discharge summary. *Proceedings - the Annual Symposium on Computer Applications in Medical Care*. 1991:136-40.

110.Finn K.M., Heffner R., Chang Y., Bazari H., Hunt D., Pickell K., Berube R., Raju S., Farrell E., Iyasere C., Thompson R., O'Malley T., O'Donnell W., Karson A. Improving the discharge process by embedding a discharge facilitator in a resident team. *Journal of Hospital Medicine (Online)*. 2011;6(9):494-500.

111.Stauffer B.D., Fullerton C., Fleming N., Ogola G., Herrin J., Stafford P.M., Ballard D.J. Effectiveness and cost of a transitional care program for heart failure: a prospective study with concurrent controls. *Archives of Internal Medicine*. 2011;171(14):1238-43.

112.Bench S., Day T., Griffiths P. Effectiveness of critical care discharge information in supporting early recovery from critical illness. *Critical Care Nurse*. 2013;33(3):41-52.

113.Duignan M., Gibbons L., O'Connor L., Denning R., Honari B., McKenna K. GPs' opinions of discharge summaries generated by advanced nurse practitioners in emergency care settings. *Emergency Nurse*. 2018.

114.Fletcher C.M., & Nuffield Provincial Hospitals Trust. Communication in medicine. . (1973).

115.*Department of Health*. Copying letters to patients: good practice guidelines. 2003 http://webarchive.nationalarchives.gov.uk/.

116.*Department of Health*. The NHS Plan: A Plan for Investment a Plan for Reform. In: HMSO, editor. London2000. Available from http://webarchive.nationalarchives.gov.uk.

117.Boaden R., Harris C. Copying letters to patients—will it happen? : Oxford University Press; 2005. Available from https://academic.oup.com/fampra/article/22/2/141/522310

118.The Academy of medical Royal Colleges. Please, write to me: Writing outpatient clinic letters to patients. *AOMRC*. 2018 https://www.aomrc.org.uk/reports-guidance/please-write-to-me-writing-outpatient-clinic-letters-to-patients-guidance/.

119.O'Driscoll B.R., Koch J., Paschalides C. Copying letters to patients: Most patients want copies of letters from outpatient clinics and find them useful. *Bmj*. 2003;327(7412):451.

120.White P. Copying referral letters to patients: prepare for change. *Patient Education & Counseling*. 2004;54(2):159-61.

121.Marzanski M., Musunuri P., Coupe T. Copying letters to patients: A study of patient's views. *Psychiatric Bulletin*. 2005;29(2):56-8.

122.Minhas R. Does copying clinical or sharing correspondence to patients result in better care? *Int J Clin Pract*. 2007;61(8):1390-5.

123.Pothier D.D., Nakivell P., Hall C.E. What do patients think about being copied into their GP letters? *Annals of the Royal College of Surgeons of England*. 2007;89(7):718-21.

124.Shee C.D. Try it and see. *Bmj*. 2008;337(a2786):p.1370.

125.Thornber M. A simple and effective communication skill. *Bmj*. 2010;337:a2324.

126.Harris E., Rob P., Underwood J., Knapp P., Astin F. Should patients still be copied into their letters? A rapid review. *Patient Education & Counseling*. 2018;101(12):2065-82.

127.Weinman J. Providing written information for patients: psychological considerations. *Journal of the Royal Society of Medicine*. 1990;83(5):303-5.

128.Main J. Copying in or copping out? *Bmj*. 2008;337(a2688):p.1369.

129.McKinstry B. Copying patients in is not as simple as it seems. *Bmj*. 2008;337(a2687):p.1369.

130.Choudhry A.J., Baghdadi Y.M., Wagie A.E., Habermann E.B., Heller S.F., Jenkins D.H., Cullinane D.C., Zielinski M.D. Readability of discharge summaries: with what level of information are we dismissing our patients? *American Journal of Surgery*. 2016;211(3):631-6.

131.Rymer J.A., Kaltenbach L.A., Anstrom K.J., Fonarow G.C., Erskine N., Peterson E.D., Wang T.Y. Hospital evaluation of health literacy and associated outcomes in patients after acute myocardial infarction. *American Heart Journal*. 2018;198:97-107.

132.Joint Commission. Provision of care, treatment, and services. . In: Edition J.C., editor. Hospital standards: PC-02.01.12, effective July 1, 2012. ; 2016. Available from

133.Buurman B.M., Verhaegh K.J., Smeulers M., Vermeulen H., Geerlings S.E., Smorenburg S., de Rooij S.E. Improving handoff communication from hospital to home: the development, implementation and evaluation of a personalized patient discharge letter. *International Journal for Quality in Health Care*. 2016;28(3):384-90.

134.Hall J.N., Graham J.P., McGowan M., Cheng A.H.Y. Using Written Instructions to Improve the Quality of Emergency Department Discharge Communication: An Interdisciplinary, Patient-Centered Approach. *Am J Med Qual*. 2018;33(2):216.

135.Lin R., Gallagher R., Spinaze M., Najoumian H., Dennis C., Clifton-Bligh R., Tofler G. Effect of a patient-directed discharge letter on patient understanding of their hospitalisation. *Internal medicine journal*. 2014;44(9):851-7.

136.McLarnon E., Walsh J., Shuilleabhain A.N. Assessment of hospital inpatient discharge summaries, written for general practitioners, from a department of medicine for the elderly service in a large teaching hospital. *Irish Journal of Medical Science (1971-)*. 2016;185(1):127-31.

137.Kergoat M.J., Latour J., Julien I., Plante M.A., Lebel P., Mainville D., Bolduc A., Buckland J.A. A discharge summary adapted to the frail elderly to ensure transfer of relevant information from the hospital to community settings: a model. *BMC Geriatrics*. 2010;10:69.

138.Dedhia P., Kravet S., Bulger J., Hinson T., Sridharan A., Kolodner K., Wright S., Howell E. A quality improvement intervention to facilitate the transition of older adults from three hospitals back to their homes. *Journal of the American Geriatrics Society*. 2009;57(9):1540-6.

139.Williams E.I., Fitton F. General practitioner response to elderly patients discharged from hospital. *Bmj*. 1990;300(6718):159-61.

140.Carlsson E., Ehnfors M., Eldh A.C., Ehrenberg A. Accuracy and continuity in discharge information for patients with eating difficulties after stroke. *J Clin Nurs*. 2012;21(1-2):21-31.

141.Raval A.N., Marchiori G.E., Arnold J.M. Improving the continuity of care following discharge of patients hospitalized with heart failure: is the discharge summary adequate? *Canadian Journal of Cardiology*. 2003;19(4):365-70.

142.Riley D.L., Krepostman S., Stewart D.E., Suskin N., Arthur H.M., Grace S.L. A mixed methods study of continuity of care from cardiac rehabilitation to primary care physicians. *Canadian Journal of Cardiology*. 2009;25(6):e187-92.

143.Yee J., Unsworth K., Suskin N., Reid R.D., Jamnik V., Grace S.L. Primary care provider perceptions of intake transition records and shared care with outpatient cardiac rehabilitation programs. *BMC Health Serv Res*. 2011;11(1):231.

144.Harel Z., Wald R., Perl J., Schwartz D., Bell C.M. Evaluation of deficiencies in current discharge summaries for dialysis patients in Canada. *Journal of multidisciplinary healthcare*. 2012;5:77.

145.Shakib S., Philpott H., Clark R. What we have here is a failure to communicate! Improving communication between tertiary to primary care for chronic heart failure patients. *Internal medicine journal*. 2009;39(9):595-9.

146.Cherlin E.J., Curry L.A., Thompson J.W., Greysen S.R., Spatz E., Krumholz H.M., Bradley E.H. Features of high quality discharge planning for patients following acute myocardial infarction. *Journal of General Internal Medicine*. 2013;28(3):436-43.

147.Marr S., Hillier L.M., Simpson D., Vinson S., Goodwill S., Jewell D., Hazzan A.A. Factors for Self-Managing Care Following Older Adults' Discharge from the Emergency Department: A Qualitative Study. *Can J Aging*. 2018:1-14.

148.Gosbee J. Communication among health professionals: human factors engineering can help make sense of the chaos. *Bmj*. 1998;316(7132):642.

149.Forster A.J., Murff H.J., Peterson J.F., Gandhi T.K., Bates D.W. The incidence and severity of adverse events affecting patients after discharge from the hospital. *Ann Intern Med*. 2003;138(3):161-7.

150.Torjesen I. Many children receive no discharge plan after admission for severe asthma. *Bmj*. 2016;355:i6403.

151.Torjesen I. Care of IBD patients compromised by poor communication between primary and secondary care. *Bmj*. 2012;344.

152.Atwal A. Nurses' perceptions of discharge planning in acute health care: a case study in one British teaching hospital. *Journal of Advanced Nursing*. 2002;39(5):450-8.

153.Sackley C.M., Pound K. Stroke patients entering nursing home care: a content analysis of discharge letters. *Clin Rehabil*. 2002;16(7):736-40.

154.Courtney E.D., Ankrett S., McCollum P.T. 28-Day emergency surgical re-admission rates as a clinical indicator of performance. *Annals of the Royal College of Surgeons of England*. 2003;85(2):75.

155.Witherington E.M., Pirzada O.M., Avery A.J. Communication gaps and readmissions to hospital for patients aged 75 years and older: observational study. *Qual Saf Health Care*. 2008;17(1):71-5.

156.Hansen L.O., Strater A., Smith L., Lee J., Press R., Ward N., Weigelt J.A., Boling P., Williams M.V. Hospital discharge documentation and risk of rehospitalisation. *BMJ Qual Saf*. 2011;20(9):773-8.

157.Donaghy E., Salisbury L., Lone N.I., Lee R., Ramsey P., Rattray J.E., Walsh T.S. Unplanned early hospital readmission among critical care survivors: a mixed methods study of patients and carers. *BMJ Qual Saf*. 2018.

158.Uppal N.K., Eisen D., Weissberger J., Wyman R.J., Urbach D.R., Bell C.M. Transfer of care of postsurgical patients from hospital to the community setting: cross-sectional survey of primary care physicians. *American Journal of Surgery*. 2015;210(4):778-82.

159.Chidwick P., Sibbald R., Hansen T., Parkes C. Managing access and flow through appropriate discharge: preventing common errors and improving processes. *Healthcare quarterly (Toronto, Ont)*. 2013;16(4):43-8.

160.Fleming M.O., Haney T.T. Improving patient outcomes with better care transitions: The role for home health. *Optimizing Home Health Care: Enhanced value and imporved outcomes*. 2013:2.

161.McLeod L.A. Patient transitions from inpatient to outpatient: where are the risks? Can we address them? *Journal of Healthcare Risk Management*. 2013;32(3):13-9.

162.Santana M.J., Holroyd-Leduc J., Southern D.A., Flemons W.W., O'Beirne M., Hill M.D., Forster A.J., White D.E., Ghali W.A. A randomised controlled trial assessing the efficacy of an electronic discharge communication tool for preventing death or hospital readmission. *BMJ Qual Saf*. 2017.

163.Yam C.H., Wong E.L., Chan F.W., Leung M.C., Wong F.Y., Cheung A.W., Yeoh E. Avoidable readmission in Hong Kong-system, clinician, patient or social factor? *BMC Health Serv Res*. 2010;10(1):311.

164.Sklar D.P., Crandall C.S., Loeliger E., Edmunds K., Paul I., Helitzer D.L. Unanticipated death after discharge home from the emergency department. *Annals of Emergency Medicine*. 2007;49(6):735-45.

165.Kripalani S., LeFevre F., Phillips C.O., Williams M.V., Basaviah P., Baker D.W. Deficits in communication and information transfer between hospital-based and primary care physicians: implications for patient safety and continuity of care. *Jama*. 2007;297(8):831-41.

166.Arora V.M., Prochaska M.L., Farnan J.M., D'Arcy V., Michael J., Schwanz K.J., Vinci L.M., Davis A.M., Meltzer D.O., Johnson J.K. Problems after discharge and understanding of communication with their primary care physicians among hospitalized seniors: a mixed methods study. *J Hosp Med*. 2010;5(7):385-91.

167.Epstein K., Juarez E., Loya K., Jo Gorman M., Singer A. Frequency of new or worsening symptoms in the posthospitalization period. *J Hosp Med*. 2007;2(2):58-68.

168.Forster A.J., Andrade J., van Walraven C. Validation of a discharge summary term search method to detect adverse events. *Journal of the American Medical Informatics Association*. 2005;12(2):200-6.

169.van Walraven C., Seth R., Austin P.C., Laupacis A. Effect of Discharge Summary Availability During Post-discharge Visits on Hospital Readmission. *Journal of General Internal Medicine*. 2002;17(3):186-92.

170.VanSuch M., Naessens J.M., Stroebel R.J., Huddleston J.M., Williams A.R. Effect of discharge instructions on readmission of hospitalised patients with heart failure: do all of the Joint Commission on Accreditation of Healthcare Organizations heart failure core measures reflect better care? *Qual Saf Health Care*. 2006;15(6):414-7.

171.Jha A.K., Orav E.J., Epstein A.M. Public reporting of discharge planning and rates of readmissions. *New England Journal of Medicine*. 2009;361(27):2637-45.

172.van Walraven C., Taljaard M., Etchells E., Bell C.M., Stiell I.G., Zarnke K., Forster A.J. The independent association of provider and information continuity on outcomes after hospital discharge: implications for hospitalists. *Journal of Hospital Medicine (Online)*. 2010;5(7):398-405.

173.Kociol R.D., Peterson E.D., Hammill B.G., Flynn K.E., Heidenreich P.A., Piña I.L., Lytle B.L., Albert N.M., Curtis L.H., Fonarow G.C. National survey of hospital strategies to reduce heart failure readmissions: findings from the Get With the Guidelines-Heart Failure registry. *Circulation: Heart Failure*. 2012:CIRCHEARTFAILURE. 112.967406.

174.Berry C. *A Study to Investigate and Design Patient Medical Education Curriculum Focusing on Drain Care Management for Use in Clinical Settings* [Ed.D.]. Ann Arbor: The University of North Carolina at Chapel Hill; 2012.

175.Bradley E.H., Curry L., Horwitz L.I., Sipsma H., Thompson J.W., Elma M., Walsh M.N., Krumholz H.M. Contemporary evidence about hospital strategies for reducing 30-day readmissions: a national study. *Journal of the American College of Cardiology*. 2012;60(7):607-14.

176.Oduyebo I., Lehmann C.U., Pollack C.E., Durkin N., Miller J.D., Mandell S., Ardolino M., Deutschendorf A., Brotman D.J. Association of self-reported hospital discharge handoffs with 30-day readmissions. *JAMA Intern Med*. 2013;173(8):624-9.

177.Retrum J.H., Boggs J., Hersh A., Wright L., Main D.S., Magid D.J., Allen L.A. Patient-identified factors related to heart failure readmissions. *Circulation: Cardiovascular Quality and Outcomes*. 2013;6(2):171-7.

178.Feltner C., Jones C.D., Cené C.W., Zheng Z.-J., Sueta C.A., Coker-Schwimmer E.J., Arvanitis M., Lohr K.N., Middleton J.C., Jonas D.E. Transitional Care Interventions to Prevent Readmissions for Persons With Heart FailureA Systematic Review and Meta-analysisTransitional Care for Persons With Heart Failure. *Ann Intern Med*. 2014;160(11):774-84.

179.Regalbuto R., Maurer M.S., Chapel D., Mendez J., Shaffer J.A. Joint Commission requirements for discharge instructions in patients with heart failure: is understanding important for preventing readmissions? *Journal of Cardiac Failure*. 2014;20(9):641-9.

180.LeClair A.M., Sweeney M., Yoon G.H., Leary J.C., Weingart S.N., Freund K.M. Patients' Perspectives on Reasons for Unplanned Readmissions. *Journal for Healthcare Quality*. 2018.

181.Gandhi T.K. Fumbled handoffs: one dropped ball after another. *Ann Intern Med*. 2005;142(5):352-8.

182.Weissman J.S., Schneider E.C., Weingart S.N., Epstein A.M., David-Kasdan J., Feibelmann S., Annas C.L., Ridley N., Kirle L., Gatsonis C. Comparing patient-reported hospital adverse events with medical record review: do patients know something that hospitals do not? *Ann Intern Med*. 2008;149(2):100-8.

183.Bell C.M., Schnipper J.L., Auerbach A.D., Kaboli P.J., Wetterneck T.B., Gonzales D.V., Arora V.M., Zhang J.X., Meltzer D.O. Association of communication between hospital-based physicians and primary care providers with patient outcomes. *Journal of General Internal Medicine*. 2009;24(3):381-6.

184.Smith K. Effective communication with primary care providers. *Pediatric Clinics of North America*. 2014;61(4):671-9.

185.Murff H.J., Forster A.J., Peterson J.F., Fiskio J.M., Heiman H.L., Bates D.W. Electronically screening discharge summaries for adverse medical events. *Journal of the American Medical Informatics Association*. 2003;10(4):339-50.

186.Moore P., Armitage G., Wright J., Dobrzanski S., Ansari N., Hammond I., Scally A. Medicines reconciliation using a shared electronic health care record. *Journal of patient safety*. 2011;7(3):148-54.

187.Viktil K.K., Blix H.S., Eek A.K., Davies M.N., Moger T.A., Reikvam A. How are drug regimen changes during hospitalisation handled after discharge: a cohort study. *BMJ Open*. 2012;2(6).

188.Cochrane R.A., Mandal A.R., Ledger-Scott M., Walker R. Changes in drug treatment after discharge from hospital in geriatric patients. *Bmj*. 1992;305(6855):694-6.

189.Munday A., Kelly B., Forrester J.W., Timoney A., McGovern E. Do general practitioners and community pharmacists want information on the reasons for drug therapy changes implemented by secondary care? *British Journal of General Practice*. 1997;47(422):563-6.

190.Grimes T., Delaney T., Duggan C., Kelly J., Graham I. Survey of medication documentation at hospital discharge: implications for patient safety and continuity of care. *Irish Journal of Medical Science*. 2008;177(2):93-7.

191.Grimes T.C., Duggan C.A., Delaney T.P., Graham I.M., Conlon K.C., Deasy E., Jago-Byrne M.C., P O.B. Medication details documented on hospital discharge: cross-sectional observational study of factors associated with medication non-reconciliation. *British Journal of Clinical Pharmacology*. 2011;71(3):449-57.

192.Jainer A.K., Noushad F., Coupe T., Mupiri C.R., Saraf A. Mind the gap-using clinical audit to minimise medication information errors at hospital discharge. *The Psychiatrist*. 2010;34(6):248-50.

193.Sands D.Z., Safran C. Closing the loop of patient care--a clinical trial of a computerized discharge medication program. *Proceedings of the Annual Symposium on Computer Application in Medical Care*. 1994 http://www.ncbi.nlm.nih.gov/pmc/articles/PMC2247756/:841-5.

194.Moore C., Wisnivesky J., Williams S., McGinn T. Medical Errors Related to Discontinuity of Care from an Inpatient to an Outpatient Setting. *Journal of General Internal Medicine*. 2003;18(8):646-51.

195.Wong J.D., Bajcar J.M., Wong G.G., Alibhai S.M., Huh J.H., Cesta A., Pond G.R., Fernandes O.A. Medication reconciliation at hospital discharge: evaluating discrepancies. *Annals of Pharmacotherapy*. 2008;42(10):1373-9.

196.Foust J.B., Naylor M.D., Bixby M.B., Ratcliffe S.J. Medication problems occurring at hospital discharge among older adults with heart failure. *Research in Gerontological Nursing*. 2012;5(1):25-33.

197.Lindquist L.A., Yamahiro A., Garrett A., Zei C., Feinglass J.M. Primary care physician communication at hospital discharge reduces medication discrepancies. *Journal of Hospital Medicine (Online)*. 2013;8(12):672-7.

198.Gattari T.B., Krieger L.N., Hu H.M., Mychaliska K.P. Medication Discrepancies at Pediatric Hospital Discharge. *Hospital Pediatrics*. 2015;5(8):439-45.

199.Sarzynski E., Ensberg M., Parkinson A., Shahar K., Brooks K., Given C. Health Information Exchange of Medication Lists: Hospital Discharge to Home Healthcare. *Home Healthc Now*. 2019;37(1):33-5.

200.Glintborg B., Andersen S.E., Dalhoff K. Insufficient communication about medication use at the interface between hospital and primary care. *Qual Saf Health Care*. 2007;16(1):34-9.

201.Kunz R., Wegscheider K., Guyatt G., Zielinski W., Rakowsky N., Donner-Banzhoff N., Müller-Lissner S. Impact of short evidence summaries in discharge letters on adherence of practitioners to discharge medication. A cluster-randomised controlled trial. Qual Saf Health Care [Internet]. 2007; 16(6):[456-61 pp.]. http://qualitysafety.bmj.com/content/16/6/456.

202.Bergkvist A., Midlov P., Hoglund P., Larsson L., Bondesson A., Eriksson T. Improved quality in the hospital discharge summary reduces medication errors--LIMM: Landskrona Integrated Medicines Management. *Eur J Clin Pharmacol*. 2009;65(10):1037-46.

203.Perren A., Previsdomini M., Cerutti B., Soldini D., Donghi D., Marone C. Omitted and unjustified medications in the discharge summary. *Qual Saf Health Care*. 2009;18(3):205-8.

204.Bertoli R., Bissig M., Caronzolo D., Odorico M., Pons M., Bernasconi E. Assessment of potential drug-drug interactions at hospital discharge. *Swiss Medical Weekly*. 2010;140:w13043.

205.Cornu P., Steurbaut S., Leysen T., De Baere E., Ligneel C., Mets T., Dupont A.G. Effect of medication reconciliation at hospital admission on medication discrepancies during hospitalization and at discharge for geriatric patients. *Annals of Pharmacotherapy*. 2012;46(4):484-94.

206.Frydenberg K., Brekke M. Poor communication on patients' medication across health care levels leads to potentially harmful medication errors. *Scandinavian Journal of Primary Health Care*. 2012;30(4):234-40.

207.Garcia B.H., Djonne B.S., Skjold F., Mellingen E.M., Aag T.I. Quality of medication information in discharge summaries from hospitals: an audit of electronic patient records. *Int J Clin Pharm*. 2017.

208.Akram F., Huggan P.J., Lim V., Huang Y., Siddiqui F.J., Assam P.N., Merchant R.A. Medication discrepancies and associated risk factors identified among elderly patients discharged from a tertiary hospital in Singapore. *Singapore Medical Journal*. 2015;56(7):379-84.

209.Carney S.L. Medication accuracy and general practitioner referral letters. *Internal medicine journal*. 2006;36(2):132-4.

210.McMillan T.E., Allan W., Black P.N. Accuracy of information on medicines in hospital discharge summaries. *Internal medicine journal*. 2006;36(4):221-5.

211.Gilbert A.V., Patel B., Morrow M., Williams D., Roberts M.S., Gilbert A.L. Providing community-based health practitioners with timely and accurate discharge medicines information. *BMC Health Serv Res*. 2012;12:453.

212.Maxwell K., Harrison J., Scahill S., Braund R. Identifying drug-related problems during transition between secondary and primary care in New Zealand. *Int J Pharm Pract*. 2013;21(5):333-6.

213.Delate T., Chester E.A., Stubbings T.W., Barnes C.A. Clinical outcomes of a home-based medication reconciliation program after discharge from a skilled nursing facility. *Pharmacotherapy:The Journal of Human Pharmacology & Drug Therapy*. 2008;28(4):444-52.

214.Bruning K., Selder F. From hospital to home healthcare: the need for medication reconciliation. *Home Healthc Nurse*. 2011;29(2):81-90.

215.Kramer J.S., Hopkins P.J., Rosendale J.C., Garrelts J.C., Hale L.S., Nester T.M., Cochran P., Eidem L.A., Haneke R.D. Implementation of an electronic system for medication reconciliation. *Am J Health Syst Pharm*. 2007;64(4):404-22.

216.Climente-Marti M., Garcia-Manon E.R., Artero-Mora A., Jimenez-Torres N.V. Potential risk of medication discrepancies and reconciliation errors at admission and discharge from an inpatient medical service. *Annals of Pharmacotherapy*. 2010;44(11):1747-54.

217.Karapinar-Carkit F., van Breukelen B.R., Borgsteede S.D., Janssen M.J., Egberts A.C., van den Bemt P.M. Completeness of patient records in community pharmacies post-discharge after in-patient medication reconciliation: a before-after study. *International Journal of Clinical Pharmacy*. 2014;36(4):807-14.

218.Borgsteede S.D., Karapinar-Carkit F., Hoffmann E., Zoer J., van den Bemt P.M. Information needs about medication according to patients discharged from a general hospital. *Patient Education & Counseling*. 2011;83(1):22-8.

219.Hohmann C., Neumann-Haefelin T., Klotz J.M., Freidank A., Radziwill R. Providing systematic detailed information on medication upon hospital discharge as an important step towards improved transitional care. *Journal of Clinical Pharmacy & Therapeutics*. 2014;39(3):286-91.

220.Bagge M., Norris P., Heydon S., Tordoff J. Older people's experiences of medicine changes on leaving hospital. *Research In Social & Administrative Pharmacy*. 2014;10(5):791-800.

221.Karapinar F., van den Bemt P.M., Zoer J., Nijpels G., Borgsteede S.D. Informational needs of general practitioners regarding discharge medication: content, timing and pharmacotherapeutic advice. *Pharmacy World & Science*. 2010;32(2):172-8.

222.Maniaci M.J., Heckman M.G., Dawson N.L. Functional health literacy and understanding of medications at discharge. *Mayo Clin Proc*. 2008;83(5):554-8.

223.Midlov P., Holmdahl L., Eriksson T., Bergkvist A., Ljungberg B., Widner H., Nerbrand C., Hoglund P. Medication report reduces number of medication errors when elderly patients are discharged from hospital. *Pharmacy World & Science*. 2008;30(1):92-8.

224.Stitt D.M., Elliott D.P., Thompson S.N. Medication discrepancies identified at time of hospital discharge in a geriatric population. *American Journal of Geriatric Pharmacotherapy*. 2011;9(4):234-40.

225.Li H., Guffey W., Honeycutt L., Pasquale T., Rozario N.L., Veverka A. Incorporating a Pharmacist Into the Discharge Process: A Unit-Based Transitions of Care Pilot. *Hospital Pharmacy*. 2016;51(9):744-51.

226.Walker P.C., Bernstein S.J., Jones J., et al. Impact of a pharmacist-facilitated hospital discharge program: A quasi-experimental study. *Archives of Internal Medicine*. 2009;169(21):2003-10.

227.Nazareth I., Burton A., Shulman S., Smith P., Haines A., Timberal H. A pharmacy discharge plan for hospitalized elderly patients--a randomized controlled trial. *Age & Ageing*. 2001;30(1):33-40.

228.Graumlich J.F., Novotny N.L., Stephen Nace G., Kaushal H., Ibrahim-Ali W., Theivanayagam S., William Scheibel L., Aldag J.C. Patient readmissions, emergency visits, and adverse events after software-assisted discharge from hospital: cluster randomized trial. *Journal of Hospital Medicine (Online)*. 2009;4(7):E11-9.

229.Mills P.R., Weidmann A.E., Stewart D. Hospital electronic prescribing system implementation impact on discharge information communication and prescribing errors: a before and after study. *Eur J Clin Pharmacol*. 2017;73(10):1279-86.
