# Supplementary material for: The Discharge Communication Study: research protocol for a mixed methods study to investigate and triangulate discharge communication experiences of patients, GPs, and hospital professionals, alongside a corresponding discharge letter sample
Source: BMC Health Serv Res. 2019 Nov 11;19:825. doi: 10.1186/s12913-019-4612-1 (PMC6849198; doi:10.1186/s12913-019-4612-1)
Supplement: Supplementary file 2 — Additional file 2. GP interview and focus group guide. [file 12913_2019_4612_MOESM2_ESM.docx]

*GP interview and focus group guide*

**Interviewer opening question:**

Please tell me about your experience(s) of patients receiving written discharge communication?

*The rest of interview or focus group will continue in a conversational manner discussing GPs views and experiences on patients receiving written discharge communication and how the discharge communication process can be improved.*

**Possible interviewer prompts:**

- What are your experiences of discharge communication as a GP?
- How do you think discharge communication can be improved?
- Please tell me your views on the discharge letters you selected for the sample?
- How would you suggest to improve these letters?
- In your opinions, is this letter suitable for a/the patient?
- What are your views on patients receiving letters?
- What do you think are important content items for good quality discharge letters?
- In your view what are the effects and outcomes of poor quality discharge letters?
