# Supplementary material for: The Discharge Communication Study: research protocol for a mixed methods study to investigate and triangulate discharge communication experiences of patients, GPs, and hospital professionals, alongside a corresponding discharge letter sample
Source: BMC Health Serv Res. 2019 Nov 11;19:825. doi: 10.1186/s12913-019-4612-1 (PMC6849198; doi:10.1186/s12913-019-4612-1)
Supplement: Supplementary file 4 — Additional file 4. Table summary of ethical issues and research team responses. [file 12913_2019_4612_MOESM4_ESM.docx]

*Table summary of ethical issues and research team responses*

| Identified Risk/ethical issue | Comment/details/response to reduce risk |
| --- | --- |
| Financial risk of participating and inequity of access | As a project focussed on communication, the research team value patient autonomy and so the patient will decide details such as time duration, location and whether they would like an advocate friend/family member present for support. Allowing patients to select the venue and location will reduce the risk of financial burden and reduce the need for patients to travel a long way which should support patients with difficulty travelling or mobility issues. Patient participants will be given a generic high street £20 thank you voucher for participating. These was discussed during PPI activity and thought to be a suitable sum. The figure of £20 is also based on the Warwick University PPI service UNTRAP* guidelines. Funding money will be available on request to reimburse any costs incurred associated with participating e.g. travel. GP practices will be compensated for their time and participation in accordance with CRN guidelines. Within the time and resource limitations of the project it is only possible to conduct interviews, focus groups and devise surveys in English. This raises potential inequity in access to the study. However, due to the small sample size and locality of the study, it is anticipated most participants will speak English. |
| Participant upset or distress | Patients will be allowed as much time as they wish to discuss their problems and can contact the research team at any time. If patients wish to report or discuss their problems with the practice, they will be referred to the correct complaint and administrative services. Where necessary, it may be suggested to the patient to discuss their concerns with their doctor. If the discharge letter is reviewed for the first time between researcher and patient [according to protocol where this is possible] the patient may become confused or upset by the discharge letter content. Although the patient should be aware of their discharge care plan and information, the letter may contain information they have not heard/seen before or forgot. In such instances, the patient will be encouraged to see their GP to answer any questions and these interviews will be encouraged to take place at the GP practice. The GPs will be aware of which patients may view their letter and available for contact/support. The research team will not answer any queries related to the letter content or provide medical advice. Queries will be referred to the relevant team. Participants can pause, stop or reschedule activities and may withdraw at any time without reason. Breaks may be taken at any point and timing of interviews will be flexible to accommodate patient needs and potential upset. |
| Patients do not individually consent to have their letter anonymously included in the sample. Patients should always have a choice to take part. | As discharge letters are being selected by GPs without direct patient consent, "opt out" posters will be displayed at participating GP practices so that patients wishing not to be involved in the study at all, even anonymously, do not have to be if they do not wish to. Information concerning “opting out” is also provided on the study invitation pack which will be sent to all patients whose letters are included in the anonymous 700 discharge letter sample. The patient’s GP practice will redact the discharge letter sample before transferral to the research team. |
| Intruding on participant time | This will be minimised by using a very short survey template and adapting the focus groups and interviews to the time/location and any other needs of the participants. |
| Safety risk to researcher during home visits | The researcher will use a “buddy system”; KW will report all travel plans and timings to a member of the supervisory team by text message upon departure and return. If the researcher does not complete data collection on time, the supervisory team will be aware and be able to follow up and escalate as necessary. The NHS lone worker policy has been consulted to increase researcher safety during this process and identify potential risks. |
| Data storage | The research team will handle data in line with NHS and Warwick University procedures. Data will be labelled with study ID codes to protect participant identities. The consent forms will be the only documents outside GP practices containing ID and other identifiable information and these will be stored separately to all other data. Any electronic files will be stored on a password protected Warwick Medical School device or computer. Hard copies of data will be stored in a data locker or locked cabinet at Warwick Medical School. |
| Confidentiality | All data will be treated as confidential within the research team. Any outputs will be anonymised. As detailed in the information sheets, quotes from participants may be used in outputs. Confidentiality will only be breached in the unlikely event that a participant reveals a safety or legal issue which may result in harm. |
| Obtaining informed consent | Verbal and written consent will be received from all participants taking part in focus groups or interview and witnessed by researcher KW. Survey participants have a choice whether or not to complete the survey and it will be made clear that completing and returning the survey means they consent to the research. Participants will be reminded during data collection that consent is voluntary and they may withdraw. Participants have the right to withdraw at any stage or decline without having to give a reason. The researcher will go through relevant phase information and ensure the participant understands what is required of them prior to commencing any and all focus groups and interviews. Any participant who indicates they lack capacity to consent will be excluded or anyone who does not demonstrate informed participation. GPs will be responsible for screening potential patient participants and therefore only selecting participants with capacity to consent. This excludes patients such as those with dementia, mental health needs and children. GPs may exclude any other patients for whom they feel participation may be particularly distressing or inappropriate e.g. end of life care. GPs should also exclude patients who have expressed a general wish not to participate in research. |

*UNTRAP: The University of Warwick and Coventry University have established an organisation called UNTRAP - Universities/Users Training and Research Action Partnership
